# Red lines and green lights: Gene therapy for inherited erythroid disorders beyond the haemoglobinopathies

**DOI:** 10.1111/bjh.70675

**Published:** 2026-07-14

**Authors:** Gaurav Joshi, Nandakumar Natarajan, Kathryn E. Dickerson, Richard A. Voit

**Affiliations:** ^1^ Department of Pediatrics, Division of Pediatric Hematology/Oncology University of Texas Southwestern Medical Center Dallas Texas USA; ^2^ Department of Pediatrics Children's Medical Center Dallas Texas USA; ^3^ Harold C. Simmons Comprehensive Cancer Center Dallas Texas USA

**Keywords:** CRISPR, gene therapy, inherited anaemia, lentiviral

## Abstract

Gene therapy is revolutionizing treatment paradigms for inherited haematological and immunological conditions. Recent successes, including the United States Food and Drug Administration (FDA) approval of gene therapy products for sickle cell disease and beta‐thalassaemia, highlight the translational path of gene therapies for erythroid‐specific disorders. In contrast, gene therapy development for other inherited erythroid disorders remains largely preclinical. Here, we examine the emerging landscape of gene therapies for inherited non‐haemoglobinopathy erythroid disorders, focusing on the status of gene therapies for Diamond–Blackfan anaemia (DBA), pyruvate kinase deficiency (PKD), X‐linked sideroblastic anaemia (XLSA), congenital erythropoietic porphyria (CEP) and congenital dyserythropoietic anaemia (CDA). We discuss the latest cellular engineering approaches being applied to developing therapies for these erythroid disorders and evolving strategies for conditioning and engraftment of modified cells. Despite the rarity of these disorders individually, several convergent biological and translational themes have emerged. Leveraging shared insights across diseases may accelerate clinical translation and broaden the curative potential of gene therapy for inherited erythroid disorders beyond the haemoglobinopathies.

## Graphical abstract

Gene therapy is revolutionizing treatment paradigms for haemoglobinopathies, establishing a translational framework for disorders that impact red blood cell development. In their paper, Joshi et al. describe the preclinical and early clinical landscape of gene therapies for non‐haemoglobinopathy erythroid disorders and highlight common thematic considerations relevant across rare disorders of erythropoiesis.

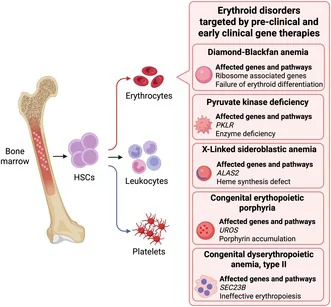

## INTRODUCTION

Anaemia is a global problem, with an estimated worldwide prevalence of 25%.^1^ While most cases of anaemia arise from nutritional deficiency or chronic disease, rare inherited forms of anaemia require lifelong treatment, often with chronic red blood cell transfusions. One of the most common inherited anaemias is beta‐thalassaemia, and the most severe form beta‐thalassaemia major requires frequent and lifelong transfusions. The morbidities associated with chronic transfusions,^2^ led to the first bone marrow transplantation for beta‐thalassaemia in 1981^3^ and stimulated interest in pursuing gene therapy for beta‐thalassaemia.^4^ Over the subsequent 40 years, alterations in vector backbone, promoter sequence and stem cell mobilization techniques^5^ culminated in FDA approval for two gene therapy products for beta‐thalassaemia, betibeglogene autotemcel in 2022 and exagamglogene autotemcel in 2024. These successes, along with concurrent FDA approvals for related products to treat vaso‐occlusive episodes associated with sickle cell disease, have established a translational pipeline for gene therapies for erythroid disorders and have motivated the next generation of gene therapy clinical trials for haemoglobinopathies. These advances and ongoing studies have been recently summarized.^6^, ^7^ While gene therapy for haemoglobinopathies has validated the feasibility of genetic correction of erythroid disorders, translation of similar strategies for other erythroid diseases has progressed more slowly. Acceleration of the clinical translation of novel therapies for these conditions is critical because despite advances in supportive care, many inherited erythroid disorders still lack safe and durable treatment options. Specific therapies like corticosteroid treatment for Diamond–Blackfan anaemia (DBA) confer significant long‐term morbidities.^8^ More broadly, chronic red blood cell transfusions treat the anaemia of erythroid disorders but are associated with morbidities including iron overload that leads to heart failure, liver dysfunction and endocrinopathies. The only currently available cure for these disorders is allogeneic haematopoietic stem cell transplantation which is associated with substantial acute and long‐term morbidity and mortality, highlighting the need for curative gene therapy approaches.

The ideal target diseases for erythroid gene therapy are genetically encoded and clinically morbid disorders in which there is prior evidence of disease amelioration following bone marrow transplantation. This review summarizes the current landscape of preclinical and early clinical efforts targeting non‐haemoglobinopathy erythroid disorders and highlights common technical and translational challenges that may guide future development. We discuss logistical and regulatory hurdles that impact the pace of clinical translation and how personalized gene therapies may overcome some of these challenges.

## GENE ADDITION AND GENE EDITING TECHNOLOGIES

Haematopoietic stem and progenitor cells (HSPCs) are attractive targets for gene therapy because they can be harvested, genetically modified ex vivo and reinfused to generate long‐term multilineage haematopoiesis (Figure 1). Disorders affecting erythropoiesis are therefore well suited to HSPC‐directed gene therapy approaches.

**FIGURE 1:**
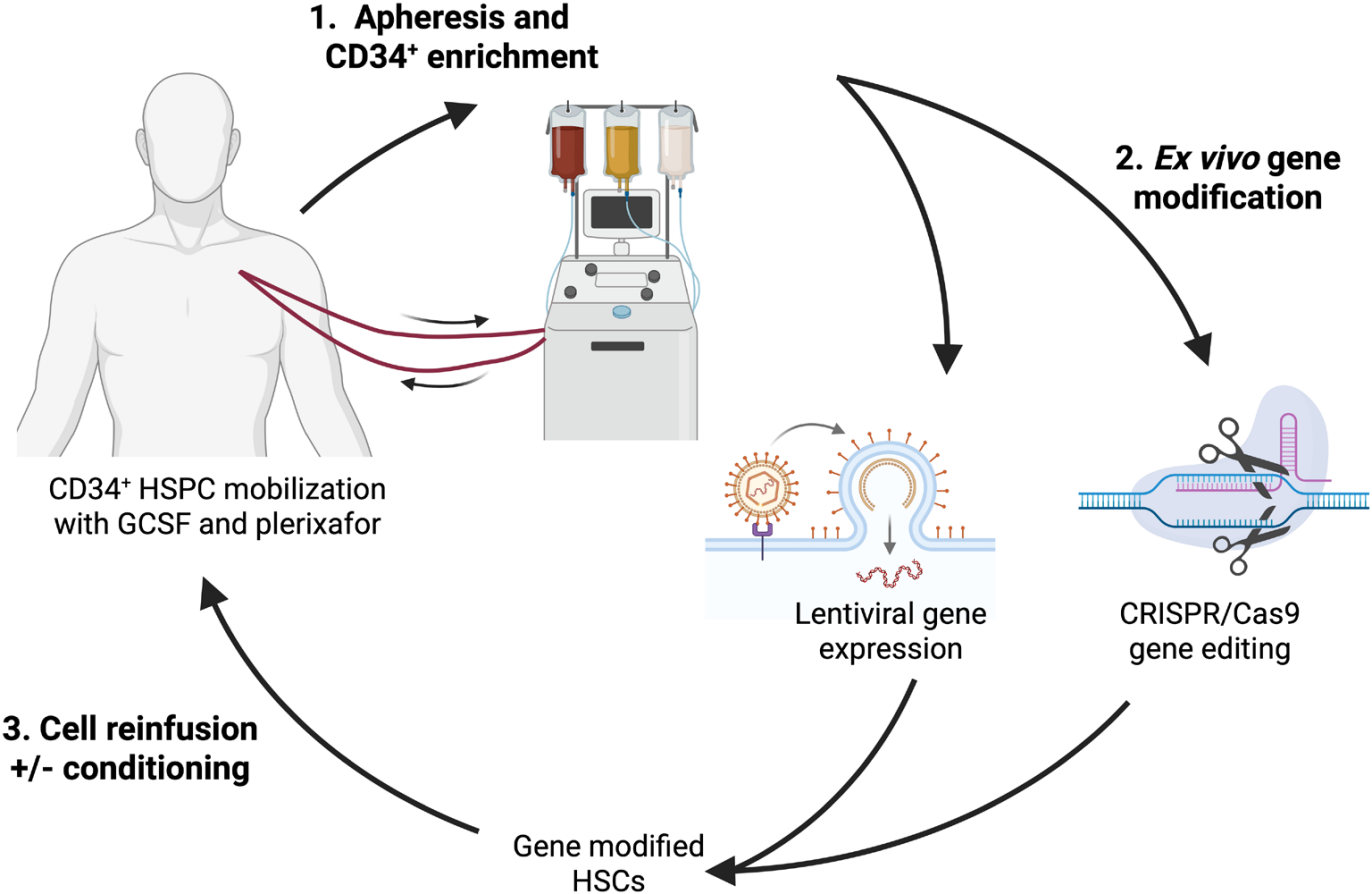
Workflow of autologous gene therapy for inherited erythroid disorders. Patient‐derived HSPCs are mobilized with granulocyte colony‐stimulating factor (GCSF) and plerixafor and collected by apheresis. HSPCs are genetically modified ex vivo using lentiviral vectors or genome‐editing approaches like Clustered Regulary Interspaced Short Palindromic Repeats (CRISPR) with CRISPR‐associated protein 9 (Cas9) to ameliorate the effects of disease‐causing mutations. The corrected cells can be transplanted directly or cryopreserved and stored before reinfusion into the patient either with or without conditioning chemotherapy to enable long‐term restoration of functional blood cell production. HSPC, haematopoietic stem and progenitor cells.

The toolbox available to achieve clinically relevant gene addition or gene editing continues to expand and has recently been comprehensively reviewed.^9^ The two major approaches being pursued in preclinical efforts for non‐haemoglobinopathy erythroid disorders are gene addition using lentiviral (LV) vectors and gene editing using CRISPR/Cas9. LV vectors are ribonucleic acid (RNA) viruses that integrate their genetic payload into the host cell (deoxyribonucleic acid) DNA following reverse transcription. LVs can modify both dividing and non‐dividing cells and can efficiently transduce HSPCs. Genomic sites of integration are enriched for transcriptionally active loci^10^ which enable long‐term expression from integrated transgene cassettes but have been associated with insertional oncogenesis in some instances. In a recent gene therapy trial for cerebral adrenoleukodystrophy, 7 of 67 treated patients developed haematological malignancy.^11^ Six patients had vector integration at *MDS1* *and* *EVI1* *complex* *locus* (*MECOM*) and the seventh at *PR*/*SET* *domain* *16* (*PRDM16*), and leukaemogenesis was attributed to oncogene activation by the strong engineered myeloproliferative sarcoma virus enhancer, negative control region deleted (MND) promoter in the vector. In contrast, a meta‐analysis of 279 patients treated with LV gene therapy for 1 of 14 other monogenic disorders revealed no genotoxic events,^12^ supporting ongoing use of LV products as a viable approach for gene addition strategies and emphasizing that promoter selection is of paramount importance.

The development and optimization of CRISPR/Cas9 tools to achieve specific genome editing outcomes is the second approach that is being pursued for gene therapies for non‐haemoglobinopathy erythroid disorders. The CRISPR/Cas9 system uses an engineered single‐guide RNA (sgRNA) to recruit Cas9 nuclease to a 20 base‐pair target sequence in the genome. Cas9 cleavage results in DNA double‐strand breaks that are repaired by one of two primary cellular processes—non‐homologous end joining (NHEJ) or homology‐directed repair (HDR). NHEJ can result in the incorporation of small base pair insertions or deletions, often modifying the reading frame of the edited gene leading to loss of functional gene expression. When an HDR template is provided, the specific intended edits can be incorporated into the site of a double‐strand break.^9^ Cas9‐directed disruption of the erythroid enhancer region of B‐*cell* *lymphoma/leukaemia 11A* (*BCL11a)* has been demonstrated to be highly effective in reactivating foetal haemoglobin (HbF) for the treatment of sickle cell disease^13^ and beta‐thalassaemia.^14^ These proof of principle studies using CRISPR/Cas9‐based gene therapy offer a framework for other inherited erythroid disorders. In addition, engineered variants of Cas9 have been developed to generate precise point mutations or specific insertions without first requiring the generation of DNA double‐strand breaks (recently reviewed in Ref. [15]). Gene therapy products that utilize adenine base editors,^16^, ^17^, ^18^ cytosine base editors^19^ and prime editors^20^ are being explored in early phase clinical trials and may be applicable to non‐haemoglobinopathy erythroid disorders in the future.^21^

Although insertional mutagenesis is not a potential concern of CRISPR‐based gene therapies as it is with LV products, the risk of genotoxicity remains to be fully elucidated through long‐term clinical follow‐up studies of CRISPR gene therapy. CRISPR/Cas9‐based approaches carry risks of off‐target editing and DNA repair–associated indel formation, underscoring the need for continued optimization of safety and specificity across gene editing platforms.

## CONDITIONING REGIMENS

Another critical consideration for all ex vivo engineered gene therapy products is how to successfully achieve engraftment of gene‐modified cells. Experience from allogeneic bone marrow transplantation demonstrates that full myeloablation prior to gene therapy is recommended for haemoglobinopathies and lysosomal storage disorders.^22^ In myeloablative conditioning regimens, complete clearance of endogenous HSPCs enables high levels of engraftment of transplanted cells.^23^ This is achieved using genotoxic chemotherapies, typically busulfan and/or cyclophosphamide, which confer significant potential short‐term toxicities, including nausea, mucositis, oral intolerance, veno‐occlusive disease, transplant‐associated thrombotic microangiopathy and infection.^24^ Long‐term toxicities include infertility, pulmonary fibrosis, endocrine dysfunction and malignancy.^25^ Treosulfan as a substitute for busulfan is associated with a lower toxicity profile^26^ and has been successfully used in allogeneic bone marrow transplants for children with non‐malignant haematological conditions^27^ and in gene therapy trials for haemophilia A.^28^

Reduced‐intensity conditioning regimens attempt to balance the risk of conditioning‐related morbidity and mortality with sufficient engraftment of gene‐modified cells. Reducing genotoxic exposure, if possible, is especially important in bone marrow failure syndromes with known cancer predisposition like Fanconi anaemia and Diamond–Blackfan anaemia.

Antibody‐based conditioning regimens strive to achieve depletion of host HSPCs while eliminating genotoxic risk. Monoclonal antibodies targeting Cluster of Differentiation 117 (CD117) on HSPCs have been developed which clear haematopoietic stem cells (HSCs) from the bone marrow niche. Conditioning with anti‐CD117 antibodies has shown promising results in allogeneic transplants for severe combined immunodeficiency^29^ and Fanconi anaemia.^30^ Antibody drug conjugates (ADC) that deliver the potent RNA polymerase II inhibitor amanitin to cells expressing CD117 have demonstrated preclinical efficacy,^31^, ^32^ but an early phase clinical trial was prematurely terminated due to a grade 5 adverse event. Evolved versions of CD117‐ADCs appear safe in non‐human primates,^33^ offering optimism for relevance in human gene therapy studies.

Finally, conditioning‐free regimens offer the potentially safest option, involving the infusion of gene‐modified HSPCs into patients who did not receive pretreatment conditioning. This has been most extensively investigated in the context of Fanconi anaemia in which preclinical studies revealed a robust proliferation advantage of gene‐modified HSPCs. This fitness advantage of gene therapy‐treated HSPCs led to testing in clinical trials.^34^, ^35^ Despite no conditioning, six of eight patients demonstrated a progressive increase in gene‐modified cells with two patients showing virtually complete engraftment and reversion of sequelae of bone marrow failure.^34^ Whether similar growth advantages of gene‐modified cells will occur in vivo in other bone marrow failure disorders remains to be seen.

## THE STATE OF GENE THERAPY FOR NON‐HAEMOGLOBINOPATHY ERYTHROID DISORDERS

The clinical successes of ex vivo gene therapies for sickle cell disease and beta‐thalassaemia provide a blueprint for the successful translation of similar therapies for other genetic haematological disorders. Here, we examine the current state of the field, highlighting the preclinical approaches to correct the red blood cell defects associated with inherited disorders of erythropoiesis (Figure 2; Table 1). Additional erythroid disorders that could be among the next wave of gene therapy targets since they are genetically encoded, clinically morbid and have been successfully treated with bone marrow transplantation include severe forms of hereditary spherocytosis, elliptocytosis and glycolytic enzyme deficiencies.

**FIGURE 2:**
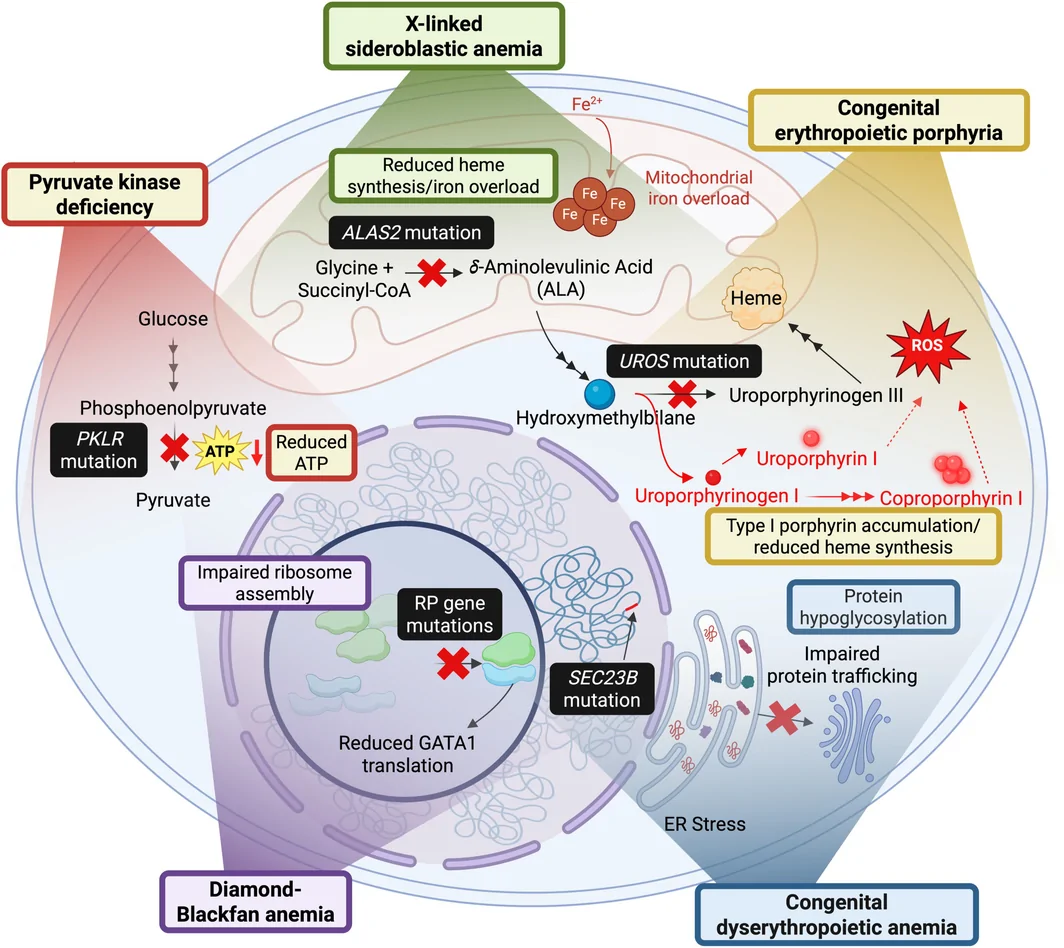
Molecular mechanisms underlying inherited red blood cell disorders. Schematic illustration of cellular pathways disrupted in inherited erythroid disorders. Diamond–Blackfan anaemia (purple highlight) is caused by mutations in one of dozens of genes that impair ribosome biogenesis, leading to reduced GATA1 translation. Pyruvate kinase deficiency (red highlight) impairs the final step of glycolysis, leading to a reduction in ATP generation. X‐linked sideroblastic anaemia (green highlight) impairs the first and rate‐limiting step of haem biosynthesis, causing reduced haem production and compensatory mitochondrial iron overload. Congenital erythropoietic porphyria (yellow highlight) involves a downstream enzyme in the haem biosynthesis pathway, causing reduced haem synthesis and toxic accumulation of Type I porphyrins. Congenital dyserythropoietic anaemia (blue highlight) impairs vesicle formation, leading to impaired protein trafficking and protein hypoglycosylation.

**TABLE 1:** Gene therapy strategies in development for inherited erythroid disorders.

Erythroid disorder	Clinical trial	Stage/year	Target	Strategy	References
Diamond–Blackfan anaemia	–	Preclinical	RPS19	Lentiviral/gene addition	Hamaguchi et al., Blood 2002
					Debnath et al., Mol Ther 2017
					Giménez et al., JCI Insight 2024
					Bhoopalan et al., Mol Ther 2025
Diamond–Blackfan anaemia	–	Preclinical	GATA1	Lentiviral (erythroid‐specific)	Voit et al., Cell Stem Cell 2025
Pyruvate kinase deficiency	NCT04105166	2020–2024	PKLR	Lentiviral	Garcia‐Gomez et al., Mol Ther 2016
					Shah et al., Blood 2022
Pyruvate kinase deficiency	NCT06422351	Planned	PKLR	Lentiviral	Shah et al., Blood 2024
Pyruvate kinase deficiency	–	Preclinical	PKLR	CRISPR/Cas9 HDR	Fañanas‐Baquero et al., Front Gen Edit 2023
Pyruvate kinase deficiency	–	Preclinical	PKLR	CRISPR/Cas9 cDNA HDR	Fañanas‐Baquero et al., Mol Ther Methods Clin Dev 2021
X‐linked sideroblastic anaemia	–	Preclinical	ALAS2	Lentiviral (erythroid‐specific)	Castruccio Castracani et al., Blood 2025
X‐linked sideroblastic anaemia	–	Preclinical	ALAS2	CRISPR/Cas9 HDR	Fang et al., Stem Cell Reports 2021
Congenital erythropoietic porphyria	–	Preclinical	UROS	Lentiviral (erythroid‐specific)	Robert‐Richard et al., Am J Hum Genet 2008
					Bedel et al., Am J Hum Genet 2012
Congenital erythropoietic porphyria	–	Preclinical	UROS	CRISPR/Cas9 HDR	Prat et al., Stem Cell Reports 2020
Erythropoietic protoporphyria		Preclinical	FECH	Gene addition/antisense oligonucleotide	Mirmiran et al., Am J Hum Genet 2019
Congenital dyserythropoietic anaemia	–	Preclinical	SEC23B	Lentiviral	Dessy‐Rodriguez et al., Blood 2021
Congenital dyserythropoietic anaemia	–	Preclinical	p60‐BBF2H7	Lentiviral	Pellegrin et al., Br J Haematol 2019

## DIAMOND–BLACKFAN ANAEMIA

Diamond–Blackfan anaemia (DBA) syndrome is a rare bone marrow failure disorder associated with severe anaemia due to impaired red blood cell maturation. Diagnostic criteria include macrocytic, reticulocytopenic anaemia and bone marrow findings of erythroblastopenia, without significant dysplasia or ringed sideroblasts. Other common findings include elevated erythrocyte adenosine deaminase (eADA) activity, elevated HbF, congenital anomalies and cancer predisposition.^36^ DBA syndrome is caused by mutations in one of more than 30 genes^37^ that result in reduced ribosome number or function and lead to impaired translation of select transcripts, most notably the erythroid master transcription factor *GATA* *binding protein* *1* (GATA1),^38^ as well as increased p53 response,^39^ and an imbalance between haem and globin synthesis.^40^ At the current time, the only effective treatment options are regular red blood cell transfusions, chronic use of corticosteroids or allogeneic haematopoietic stem cell transplantation.

Until recently, preclinical gene therapy approaches have focused on the most common genotype *Ribosomal Protein S19* (*RPS19*) since approximately 25% of genetically resolved DBA patients carry mutations in *RPS19*. Early work demonstrated that enforced expression of RPS19 by LV transduction into *RPS19*‐mutated DBA patient HSPCs improved erythroid output in vitro.^41^ Further efforts developed an LV with *RPS19* expression driven by the clinically relevant elongation factor 1a short (EFS) promoter.^42^ In an inducible mouse model of Rps19 knockdown,^43^ treatment of c‐kit^+^ bone marrow cells with EFS‐RPS19 resulted in stable long‐term immune reconstitution, rescue of the lethal bone marrow failure and improvement in erythroid output.^44^ In parallel, Bhoopalan et al. developed a clinically relevant self‐inactivating LV containing the same EFS promoter to drive *RPS19* expression in a CRISPR‐derived human HSPC model of *RPS19*‐mutated DBA. CRISPR treatment of primary human CD34^+^ HSPCs resulted in a heterogenous population of *RPS19*‐edited and non‐edited alleles. Without EFS‐RPS19 rescue, there was a profound selective disadvantage of cells with *RPS19* edits, but LV treatment was sufficient to promote survival and erythroid differentiation in vivo following xenotransplantation.^45^ Similarly, LVs using either the EFS promoter or the clinically relevant phosphoglycerate kinase (PGK) promoter led to increased erythroid production from *RPS19*‐mutated primary DBA CD34^+^ HSCs in vitro. Further characterization of the PGK‐RPS19 LV demonstrated increased human erythroid differentiation in vivo following xenotransplantation.^46^

Additionally, there is a selective advantage conferred to *RPS19*‐mutated HSCs upon LV rescue.^47^ This preclinical observation is consistent with case reports demonstrating somatic reversion of DBA mutations in which individual clones spontaneously correct the pathogenic mutation. The growth advantage of revertant clones is sufficient to restore the erythroid compartment and lead to spontaneous treatment independence.^48^, ^49^ This may enable clinical gene therapy studies in DBA to target lower vector copy number (VCN) goals, leading to potentially safer products. Moreover, because of the selective advantage of gene‐modified HSCs, myeloablative conditioning regimens may not be required. Testing of reduced‐intensity, antibody‐based or conditioning‐free regimens may be on the horizon for DBA gene therapy.

The consistent stimulation of erythroid production from all three groups using RPS19 LV gene addition provides optimism that this approach will lead to clinically meaningful improvements in anaemia in clinical trials for patients with *RPS19*‐mutated DBA, and these trials are expected to begin soon. Despite the preclinical success of this approach, however, its clinical use will be limited to patients with *RPS19*‐mutated DBA. For the ~75% of DBA patients with alternative genotypes, genotype‐specific vectors would need to be developed and tested, which presents both practical and financial obstacles to gene replacement strategies for non‐RPS19‐mutated DBA. These limitations motivated the development of a genotype‐agnostic strategy based on augmenting GATA1 expression. This approach is based on the observation that GATA1 translation is specifically impaired in DBA^38^ due to the structure of its 5′UTR.^50^ Rather than using a constitutively expressing promoter like EFS or PGK, we developed a vector that is expressed only in developing erythrocytes and not in HSCs. This feature is essential because unregulated GATA1 expression in HSCs impairs stem cell function and drives exclusive erythroid lineage commitment.^51^ We defined the regulatory elements responsible for erythroid‐specific expression of GATA1, which we called the human GATA1 enhancer (hG1E) element. The hG1E element drives robust expression of GATA1 only in developing erythroid progenitors, and this regulated lineage‐restricted expression was sufficient to stimulate massively increased red blood cell production from primary HSCs collected from 12 patients with DBA of varying genotypes. Importantly, HSCs treated with the hG1E‐GATA1 vector did not express GATA1, and there was no impairment of HSC engraftment upon xenotransplantation. The integration profile of this vector was polyclonal without predilection for integration near oncogenes.^52^ These preclinical efficacy and safety data support the clinical translation of this genotype‐agnostic approach to gene therapy for DBA.

Both the RPS19 rescue and regulated GATA1 strategies are approaching clinical testing in Phase I/II studies. Because of the limited number of patients that can be enrolled in early phase gene therapy studies, pursuing both gene therapy products in parallel will hasten the broader availability of gene therapy options for individuals with DBA.

## PYRUVATE KINASE DEFICIENCY

Pyruvate kinase deficiency is the most common inherited non‐spherocytic haemolytic anaemia with an estimated prevalence of ~1:20 000–1:100 000 worldwide. More than 260 unique mutations in the *PKLR* gene have been described^53^ and cause haemolytic anaemia of varying clinical severity. RPK, the protein product of *PKLR*, catalyses the final step in glycolysis and deficiency preferentially impairs terminally differentiated erythroid precursors that rely on glycolysis for adenosine triphosphate (ATP) generation. Chronic blood transfusions are the historical first‐line therapy although the targeted *PKLR* activator mitapivat has shown efficacy in reducing transfusion burden in many patients with PKD.^54^ Patients with biallelic nonsense mutations are unlikely to respond to mitapivat so for these individuals treatment options are limited. This led to the preclinical development of an LV providing constitutive expression of RPK which was shown to correct glycolytic abnormalities and rescue anaemia in PKD mice.^55^ LV treatment of human CD34^+^ HSCs did not impair engraftment upon xenotransplantation,^56^ suggesting no detrimental impact on HSC function. Together, these data formed the basis for the first‐in‐human gene therapy trial for PKD using the LV product RP‐L301 (ClinicalTrials.gov: NCT04105166) that has recently completed. Interim results revealed no serious adverse events and robust and sustained efficacy at 36 months post‐treatment of four patients who remain transfusion independent.^57^, ^58^ These results have led to a planned Phase II study (ClinicalTrials.gov: NCT06422351).

In addition to LV gene therapies, CRISPR‐mediated specific gene correction approaches have been explored for PKD. CD34^+^ HSPCs from PKD patients were treated with Cas9 protein complexed with mutation‐specific sgRNAs and a single strand oligonucleotide HDR template. The efficiency of targeted correction was low and there was evidence of non‐specific insertions and deletions near the CRISPR cut site,^59^ but these results demonstrate the preclinical feasibility of personalized gene editing in PKD. In addition, HDR‐mediated insertion of full‐length *PKLR* complementary DNA (cDNA) at the endogenous locus offers a mutation‐agnostic strategy capable of correcting a broad range of *PKLR* variants.^60^ Optimization with newer CRISPR tools including base editors or prime editors could improve the editing specificity and augment the clinical relevance of this strategy.

## X‐LINKED SIDEROBLASTIC ANAEMIA

X‐linked sideroblastic anaemia (XLSA) is the most common congenital sideroblastic anaemia and affects hemizygous males and some heterozygous females, due to skewed X inactivation.^61^ XLSA is caused by mutations in *5‐aminolevulinate synthase* (*ALAS2*), an erythroid‐restricted enzyme that catalyses the first and rate‐limiting step of haem biosynthesis. Affected individuals are characterized by a microcytic, hypochromic anaemia due to impaired haem production and progressive, systemic iron overload due to ineffective erythropoiesis and transfusional iron. Erythroid precursors in the bone marrow deposit excess iron in perinuclear mitochondria forming the pathognomonic ringed sideroblasts.^62^ Recent mouse models have recapitulated the human phenotype using either CRISPR/Cas9‐mediated knock‐in of clinical mutations^63^ or conditional floxed *Alas2* knockout by inducible Cre recombinase expression.^64^ These mouse models have enabled the preclinical study of novel gene therapies. LV delivery of human *ALAS2* under the control of the human beta‐globin locus control region (LCR) was shown to achieve erythroid‐specific transgene expression. *ALAS2* LV treatment and transplantation of lineage‐negative HSCs from *Alas2* conditional knockout mice rescued the lethal anaemia and restored parameters of iron dysregulation and mitochondrial dysfunction seen with Alas2 loss. This effect was observed only at VCN >0.6, highlighting the dose dependency of ALAS2 rescue in mouse haematopoiesis.^64^ The impact of erythroid‐specific ALAS2 LV on human erythropoiesis from healthy donors or XLSA patients and the safety profile in human HSCs remains to be seen.

*ALAS2* gene correction by CRISPR‐mediated homology‐directed repair has shown preclinical promise as an alternative gene therapy approach for XLSA. XLSA patient‐derived induced pluripotent stem cells and mobilized peripheral blood CD34^+^ HSPCs were nucleofected with Cas9 messenger RNA (mRNA), synthetic guide RNA targeting *ALAS2* and a single‐stranded DNA oligonucleotide HDR template. Editing efficiency approached 50% and was associated with increased *ALAS2* expression, restored haem biosynthesis and improved erythropoiesis in vitro. There was also preserved function of modified HSCs in vivo noted on secondary xenotransplantation.^65^ While this approach provides a precise HDR‐mediated correction of the endogenous gene, widespread utility may be limited since more than 100 unique variants in *ALAS2* have been described to cause XLSA, each of which would require design and validation of CRISPR repair machinery.^66^

## CONGENITAL ERYTHROPOIETIC PORPHYRIA

Congenital erythropoietic porphyria (CEP) is another genetic metabolic disorder that impairs haem biosynthesis and primarily causes dermatological phototoxicity and variable, but often severe haemolytic anaemia. More than 50 distinct mutations in *uroporphyrinogen synthase* (*UROS*) have been identified in patients with CEP.^67^ One mutation associated with a severe clinical phenotype is P248Q, and this mutation in mice phenocopies the human disease,^68^ enabling preclinical evaluation of gene therapies for CEP. HSCs from the CEP mouse were treated ex vivo with an LV containing human *UROS* cDNA driven by the erythroid‐specific, chimeric HS‐40/Ankyrin‐1 promoter. Upon transplantation, the *UROS* LV treated mouse HSCs engrafted and rescued parameters of CEP including increased UROS enzymatic activity, reduced porphyrin accumulation and improved anaemia. There was also a selection for gene‐corrected erythroid cells in vivo, highlighting the survival advantage of erythroid progenitors with restored UROS expression.^69^ As with Fanconi anaemia complementation group A (*FANCA*) replacement for Fanconi anaemia and *RPS19* replacement for DBA, this selective advantage of gene‐modified cells may impact the VCN required to achieve clinical benefit and may inform conditioning regimen requirements. The same erythroid‐specific LV was used to restore UROS activity in CEP patient‐derived induced pluripotent stem cells (iPSCs), and in vitro erythroid differentiation and metabolic parameters of CEP were rescued.^70^

Targeted gene correction using CRISPR‐mediated HDR has also been demonstrated as a potential approach for gene therapy for CEP. CEP patient‐derived iPSCs were treated by nucleofection of plasmid‐encoded Cas9, sgRNAs designed to target only the specific CEP causing mutations, and a single‐stranded oligonucleotide HDR template. This approach not only led to low‐level specific gene correction but also induced high levels of non‐specific insertions and deletions at the Cas9 cut site. Further optimization of the strategy is required prior to clinical utility. Even then, since compound heterozygous mutations are often seen in this autosomal recessive disorder, a very personalized approach would be essential to correct the specific mutations in any individual patient.^71^

Additional forms of porphyria including erythropoietic protoporphyria (EPP) are caused by mutations in other enzymes of the haem biosynthesis pathway. Although severe anaemia is not a common clinical manifestation of EPP, reduced *ferrochelatase* (*FECH*) activity in erythroid progenitors leads to free protoporphyrin IX which accumulates in the skin and liver causing photosensitivity and liver disease. Nucleotide‐based and LV gene therapies aiming to restore FECH activity in erythroid progenitors are being explored to treat the hepatic and dermatological manifestations of EPP.^72^ This approach reduces toxic protoporphyrin levels and systemic sequelae of EPP, highlighting the therapeutic potential of erythroid‐based gene therapies beyond the treatment of severe anaemia. Gene therapy strategies to achieve erythroid expression of transgenes to serve as delivery vehicles to treat haemophilia, metabolic disorders or other diseases arising from non‐erythroid cells remain an important frontier.^73^

## CONGENITAL DYSERYTHROPOIETIC ANAEMIA

Congenital dyserythropoietic anaemia (CDA) is a class of rare disorders characterized by morphological aberrancies of erythroid progenitors and ineffective erythropoiesis. Individuals present with varying degrees of haemolytic anaemia with inappropriately low reticulocyte response. There is bone marrow hypercellularity with increased but dysplastic erythroid progenitors.^74^ CDA type II is the most common form and is caused by biallelic *SEC23* *homolog* *B* (*SEC23B*) mutations, which motivated the development of LVs expressing *SEC23B* driven by the PGK promoter. LV delivery of PGK‐SEC23B into CRISPR‐generated *SEC23B* knockout human CD34^+^ HSPCs restored erythroid maturation, with decreased dysmorphic progenitors.^75^ Impact of this treatment on HSCs and vector persistence in in vivo models will be essential to characterize further prior to clinical translation.

Another preclinical gene therapy approach for CDA‐II is based on the observation that the SEC23B paralog, SEC23A shares high sequence similarity and overlapping function but is downregulated during erythroid differentiation.^76^ *SEC23A* expression is regulated by the N‐terminal fragment of the endoplasmic reticulum stress transducer protein CREB3L2 (also known as BBF2H7). LV expression of this transcriptionally active peptide, called p60‐BBF2H7, was shown to upregulate *SEC23A* expression during erythropoiesis, stimulate growth of CDA‐II patient erythroid progenitors and partially reverse the membrane protein hypoglycosylation that is observed in CDA‐II erythroid cells.^77^ However, the impact of p60‐BBF2H7 expression in other lineages and in particular in HSCs is not known and will need to be closely characterized before this can become a clinically tractable approach.

Additionally, while CDA‐II is the most common form, the clinical manifestations are often much milder than other forms, and gene therapy with its associated potential toxicities may not be warranted for most CDA‐II patients. Bone marrow transplantation is often pursued in CDA‐IV, the most severe form, but only 10 definitive cases have been described,^78^ limiting the potential reach of gene therapy for CDA‐IV.

## OTHER CONDITIONS WITH ERYTHROID DYSFUNCTION

We have focused this review on gene therapies for non‐haemoglobinopathy, erythroid‐specific disorders, but other haematological conditions that impact multiple lineages and have erythroid dysfunction as a feature are worth a brief mention here. Fanconi anaemia and Shwachman–Diamond syndrome are bone marrow failure syndromes that impact all three haematopoietic lineages and include anaemia as a clinical feature. As discussed above, conditioning‐free autologous bone marrow transplants following gene therapy with LV expressing *FANCA* have shown acceptable safety profiles and evidence of efficacy, particularly in patients receiving the highest gene therapy cell doses.^34^ Shwachman–Diamond syndrome gene therapy is not yet as mature but has focused on antisense oligonucleotides to regulate gene splicing, and CRISPR base editing and prime editing in preclinical contexts.^79^

Deficiency of adenosine deaminase 2 (DADA2) is a systemic inflammatory and vasculitic condition that sometimes manifests with pure red cell aplasia, with many overlapping features with DBA. LV gene addition of *ADA2* has shown preclinical promise with improved haematopoietic and immunological parameters of DADA2 patient‐derived HSCs.^80^

## COMMON THEMES IN ERYTHROID GENE THERAPY DEVELOPMENT

Because of the rarity of these inherited erythroid disorders individually, common themes and shared insights across disorders are critical to recognize and leverage in the clinical translation of these gene therapies. Erythroid‐specific transgene expression is a critical aspect of some of these preclinical gene therapies, including XLSA, CEP and the GATA1‐directed strategy for DBA. The desired timing and level of optimal gene expression during erythropoiesis inform the selection or development of novel erythroid‐specific promoters. Limiting transgene expression to the impacted erythroid lineage may improve the safety profile of these approaches, avoiding the oncogene activation seen in studies using strong, constitutively active promoters like MND. To date, erythroid‐specific regulatory elements used in preclinical gene therapy vectors have included the beta‐globin LCR, human GATA1 enhancer and the HS‐40/Ankyrin‐1 promoter. In addition, massively parallel screening approaches using DNAse hypersensitivity sites in haematopoietic lineages have uncovered dozens of erythroid‐specific putative regulatory elements, some of which outperformed beta‐globin LCR‐driven erythroid‐specific gene therapy vectors.^81^ These sequences are highly enriched for binding motifs of erythroid transcription factors GATA1, TAL1, KLF1 and NFIX, and further experimental and in silico evaluation of these sequences may lead to engineered enhancer elements that are tunable to be active only at specific stages of erythroid maturation.

In other disorders such as Fanconi anaemia and *RPS19*‐mutated DBA, constitutive transgene expression in all haematopoietic stem cells including HSPCs leads to a selective growth advantage that influences target VCN goals and the selection of optimal conditioning regimens. Reducing the intensity of conditioning, or eliminating it altogether, greatly reduces the short‐ and long‐term toxicities associated with gene therapy, and might be most relevant in *RPS19*‐mutated DBA and CEP, though it remains to be seen whether partial correction in PKD, CDA and XLSA would be sufficient to support reduced‐intensity conditioning regimens. More broadly for all disorders discussed here, a shared challenge is defining the minimal VCN required for therapeutic benefit while minimizing the risk of insertional mutagenesis.

Finally, genetic heterogeneity with hundreds of possible mutations in a causative gene, as seen across the board in the inherited erythroid disorders discussed here, influences the selection of the optimal gene therapy platform. Until a scientific and regulatory pipeline for personalized gene therapy is established, CRISPR‐based corrections of these disparate mutations are unlikely to become widely available clinical options.

## DISCUSSION

From the first gene therapy studies in the 1980s to the first FDA approval of a LV gene therapy product to treat an inherited disorder in 2022, the prospect of genetic cures for haematological and immunological diseases has been a tantalizing treatment modality. The evolution of vector design, conditioning regimens and post‐transplant clinical management has served to improve safety and optimize efficacy such that gene therapy is a viable curative option for an increasing number of diseases. In contrast to the haemoglobinopathies, there are many fewer patients with other inherited disorders of erythropoiesis, so despite robust preclinical evidence of benefit, clinical translation of these therapies has lagged. A primary driver is the cost to conduct clinical gene therapy studies. Recent estimates based on 11 gene therapy products that are either FDA approved or in late stages of clinical testing report a direct cost of more than US$500 million from the initiation of phase I trials to FDA approval. Capitalized and risk adjusted cost estimates balloon to almost US$2 billion.^82^ Further accounting for the preclinical research and development costs, which have been estimated to range between 9.6%^83^ and 42.9%^84^ of total capitalized costs, drives the price tag even higher.

In vivo delivery of gene therapy payloads would address some of these cost considerations. In vivo gene therapy eliminates the need for specialized and costly ex vivo cellular manufacturing and bypasses the need for conditioning chemotherapy which in turn requires prolonged and expensive hospitalization until engraftment of engineered cells. At least 67 clinical trials of in vivo gene therapy using adenovirus, adeno‐associated virus and lipid nanoparticle delivery vectors are underway,^16^ focusing primarily on tissues like the brain, liver and heart that cannot be modified ex vivo. However, preclinical studies in mice^85^ and non‐human primates^86^ demonstrate the feasibility of in vivo gene therapy for haemoglobinopathies. Additionally, a recent study has demonstrated the feasibility of in vivo LV HSPC gene therapy in Fanconi anaemia where the selective advantage of modified cells achieves therapeutic benefit despite modest initial gene transfer efficiencies. This feature may make in vivo gene therapy especially attractive for diseases in which corrected haematopoietic stem cells can outcompete their uncorrected counterparts.^87^

An important unknown across gene therapies for inherited erythroid disorders is the level of correction that is required to achieve clinical benefit. While thresholds analogous to HbF induction for sickle cell disease remain largely undefined, preclinical PKD studies suggest that correction of ~20%–30% of haematopoietic stem cells may be sufficient to improve erythroid parameters, indicating that full correction may not be required.^56^ Spontaneous reversion events in DBA suggest that even lower levels of gene‐modified cells may be sufficient for therapeutic benefit in some disorders.^48^, ^49^

Beyond mutation‐specific gene correction, there is growing interest in developing universal therapeutic strategies that target shared downstream pathways of ineffective erythropoiesis. In disorders such as DBA, where ribosomal dysfunction converges on impaired erythroid maturation, modulation of common erythroid regulatory or stress‐response pathways may offer an alternative strategy to gene replacement, potentially enabling broader applicability across genetically heterogeneous disease subtypes. Approaches to regulate p53 response, haemoglobin imbalance, and integrated stress response may be applicable across erythroid disorders, but specific gene therapy‐based strategies to achieve this have not yet been developed. Universal gene therapies like our GATA1 approach for DBA need to be carefully evaluated in preclinical studies, since the underlying causative mutations do not always drive mechanistically identical pathologies. For example, single‐cell profiling of DBA patient samples from patients with different RP gene mutations revealed varied impacts on GATA1 with the most profound downregulation of GATA1 target genes seen in *RPS19* patient samples with unchanged or even upregulation of GATA1 target genes in some erythroid progenitor populations from patients with *RPL5* and *RPL11* mutations.^88^ Despite these observations, we showed stimulation of erythroid maturation by regulated GATA1 expression across patient genotypes, including *RPL5* and *RPL11*.^52^ A specific focus on deepening our mechanistic understanding of the lineage‐ and stage‐specific pathologies of causative mutations is critical for the successful development and implementation of broader gene therapies targeting common mechanisms.

Another important frontier in gene therapy for non‐haemoglobinopathy erythroid disorders is the prospect of personalized CRISPR‐mediated gene correction. Because dozens and in some cases hundreds of specific mutations in an individual gene can cause similar clinical phenotypes, an off‐the‐shelf CRISPR correction strategy is unlikely to be feasible for erythroid disorders beyond sickle cell disease. However, the recent success of the ‘N‐of‐1’ in vivo CRISPR base editing correction of the ultra rare inborn error of metabolism, carbamoyl‐phosphate synthetase 1 (CPS1) deficiency,^17^ suggests that wider development of personalized gene therapies for inherited disorders may be on the horizon.^89^, ^90^ The rapid evolution of gene editing technologies, vector engineering and conditioning strategies suggests that gene therapy for rare inherited erythroid disorders is approaching a translational inflection point. Realizing this potential will require coordinated efforts between academic investigators, industry partners and regulatory agencies to overcome economic and logistical barriers that disproportionately affect ultra rare diseases.

While curative haematopoietic stem cell transplantation remains an option for a subset of patients, the risks associated with donor availability, graft‐versus‐host disease and transplant‐related toxicity limit its widespread application. Accordingly, the ideal patient for initial gene therapies for non‐haemoglobinopathy erythroid disorders is an adolescent or young adult with genetically confirmed erythroid disease. He or she has severe disease manifestations including transfusion dependency or intolerable treatment side effects, but with low iron burden and minimal systemic toxicities. There should be no evidence of myelodysplasia in the bone marrow. The ideal patient does not have an unaffected, matched sibling donor and should not have previously undergone bone marrow transplantation. It is also critical to assess psychosocial factors that impact patient readiness and resilience.^91^ Following demonstration of safety and efficacy in initial cohorts of patients, younger patients or those who have matched sibling donors but would prefer gene therapy to eliminate risks of graft‐versus‐host disease will be good candidates for gene therapy.

In a similar way, the ideal gene therapy approach for these disorders will evolve over time. Initially, the best gene therapy will be the one that is available as there is a growing desire from patients and caregivers in advocacy groups like the Diamond Blackfan Anemia Foundation for better treatment modalities like gene therapy. Currently, there is equipoise between LV and CRISPR‐mediated gene therapy platforms, but longer clinical follow‐up of patients who have undergone gene therapy, and the continued evolution of gene editing tools may one day shift that balance. The in vivo delivery of HSC‐targeted gene editing machinery to achieve highly efficient, on‐target, patient mutation‐informed CRISPR base editing or prime editing is the ultimate goal. This will require a concerted effort among scientists, clinicians, federal and private funding agencies, industry partners, regulators and payors to ensure that the exciting progress of the past decade is not stifled and will become curative, accessible therapies for all patients.

Although that lofty goal has not yet been realized, advances in gene therapy described here have created realistic opportunities to correct the underlying molecular defects in many erythroid disorders. Preclinical studies across multiple diseases including those reviewed here demonstrate that genetic correction of HSCs can restore erythropoiesis and ameliorate disease phenotypes. Several common themes have emerged from these efforts that require careful consideration to enable successful clinical translation. Continued improvements in vector design, genome editing technologies and safer conditioning strategies are likely to accelerate clinical translation. Although these diseases individually affect relatively small patient populations, the lessons learned from their study will inform the broader development of gene therapies for haematological disease and may ultimately expand the curative potential of erythroid gene therapy beyond the haemoglobinopathies.

## AUTHOR CONTRIBUTIONS

**Richard A. Voit:** Conceptualization; writing – original draft; writing – review and editing; visualization; supervision; funding acquisition. **Gaurav Joshi:** Conceptualization; visualization; writing – review and editing; writing – original draft. **Kathryn E. Dickerson:** Writing – review and editing. **Nandakumar Natarajan:** Conceptualization; writing – review and editing.

## FUNDING INFORMATION

RAV is supported by the NCI/NIH (grant K08CA286756), the Edward P. Evans Foundation, Alex's Lemonade Stand Foundation (22‐27254), the V Foundation and the Cancer Prevention and Research Institute of Texas (RR240039), and is a Horchow Family Scholar.

## CONFLICT OF INTEREST STATEMENT

RAV is a named inventor on patent PCT/US2020/036600 filed by Boston Children's Hospital related to regulated GATA1 gene therapy for DBA. The authors have no other conflicts of interest to declare.

## Data Availability

Data sharing not applicable to this article as no datasets were generated or analysed during the current study.
